# An empirical assessment of the influence of digital transformation on sports corporate sustainability

**DOI:** 10.1371/journal.pone.0297659

**Published:** 2024-04-18

**Authors:** Haixia Wang, Laibing Lu, Yidan Fu, Qiuying Li

**Affiliations:** 1 Handan Univeraity, College of Physical Education, Handan, China; 2 School of Physical Education and Sports Science, Fujian Normal University, Fuzhou, China; 3 Star for PH.D in Sport Fitness Science Gdansk University of Physical Education and Sports, Gdansk, Poland; 4 Jiangsu Normal University, Physical Education Institute, Xuzhou, China; 5 Department of Sports Rehabilitation, Hunan University of Medicine, Huaihua, China; King Abdulaziz University, SAUDI ARABIA

## Abstract

The trend of digital transformation fosters enterprise change, helps cultivate enterprises’ own competitive advantages and is crucial to the advancement of sports enterprises’ sustainable development in the framework of the emerging digital economy as a national strategy. However, there have been few empirical studies on the microlevel of digital transformation and its impact on the sustainability of sports organizations. Therefore, the sustainable growth dynamic model is used to construct indicators of corporate sustainability by referencing 48 sports corporations listed on Shanghai and Shenzhen A-shares markets and the New Third Board in China from 2012 to 2021. The intrinsic relationship between digital transformation and the sustainable development of sports enterprises and the underlying mechanism of action are explored by constructing a panel fixed effects model, a chain mediating effects model, and a panel threshold model. The most important contribution is as follows: To provide a useful reference for analyzing enterprise digital transformation, a more complete indicator indicating the extent of corporate digital transformation is built. The micro viewpoint broadens our awareness of sustainable development in sports organizations and deepens our understanding of the interaction model between sustainable development and enterprise digital transformation. This study provides methodical evidence and insights for an accurate understanding of digital transformation for sustainable enterprise development, looking into the "black box" of the mechanism between digital transformation and sustainable business development. The results show that digital transformation significantly aids sports enterprises in their pursuit of long-term sustainability. Heterogeneity tests demonstrate the pivotal role of digital transformation in advancing the sustained growth of sports firms and high-tech sports enterprises situated in the eastern region of China. Regarding transmission mechanisms, the chain mediating effect of enterprises’ digital transformation on improved technological innovation and TFP, which in turn promote long-term business growth, has yet to be validated. Further examination exposes that within the context of the correlation between digital transformation and the sustainability of corporations, there is a single threshold effect based on financing restrictions and operational costs and a double threshold effect based on operational efficiency.

## 1 Introduction

The digital economy’s significance as both a stabilizing force and an engine of economic growth has garnered increasing attention in light of the recent wave of scientific, technological, and industrial advancements. This trend presents fresh prospects for nations while simultaneously presenting enterprises with unparalleled challenges. Given the swift growth of the digital economy, China is embarking on a new era characterized by ’digitalization.’ The profound amalgamation of digital technology and traditional sectors has given rise to considerable momentum. As of the close of 2021, China’s digital economy was projected to have a valuation of 45.5 trillion yuan, equivalent to approximately 39.8% of the nation’s GDP. This phenomenon signifies a fresh impetus for China’s pursuit of high-quality economic growth and lends momentum to the sustainable development of enterprises [1]. Therefore, exploiting the windfall of digital development and accelerating digital transformation are essential strategic tasks for China’s endeavor to build a strongly networked country and a digital China as part of the 14th Five-Year Plan.

During the successful hosting of the Beijing Winter Olympics in China, digital technologies such as intelligent broadcasting, smart wear, energy-saving and emission-reducing technologies, cloud computing, and online retailing were deeply integrated. These technologies penetrated all aspects of the event, facilitating athletes’ participation, spectators’ viewing, and the holding of events, this underscores the pivotal role of digital technologies in shaping production practices within the sports industry and underscores the seamless convergence of the digital economy and the real economy within the sports sector [2]. International Olympic Committee President Bach praised Utilizing digital technology during the Beijing Winter Olympics for establishing a new technical standard for the Games and accelerating their digital transformation [2]. Therefore, digital innovation in the sports industry during the era of the digital economy is a significant aspect of growth related to the development of China’s sports industry [3], offering a new stimulus for the sports industry to achieve the development goals of more than 5 trillion yuan by 2025 and becoming a pillar industry of China’s national economy by 2035. The implementation and use of digital transformation by sports companies aim to transform and upgrade traditional business models, organizational structures, and marketing; facilitate the internal amalgamation and external extension of both existing and novel resources and capabilities; trigger business activities, capability turnover, and business model transformation to improve efficiency and innovation; and offer new opportunities for sustained growth [4, 5]. Much of the existing research on digital transformation and sustainability has focused on macrolevel sustainability [6] and industry-level sustainability [7], but little attention has been given to firm-level sustainability, particularly in the area of sustainability in sports firms. As a result, this study fills a gap in the literature by using quantitative analysis to examine the connection between digital transformation and the long-term growth of sports companies.

## 2 Literature review

### 2.1 The connotations and factors influencing sustainable enterprise development

Corporate sustainability, an ecological concept with economic implications, pertains to the objective of achieving long-term economic well-being, safeguarding the environment, and enhancing social welfare by striking a balance among economic, environmental, and social advantages while fulfilling immediate requirements [8]. This concept includes economic sustainability, which refers to the need for companies to actively explore innovative business models, adapt to changes in the market, and ensure that long-term profitability matches business growth [9]. Environmental sustainability indicates that companies should actively implement environmental measures to protect natural resources, reduce ecological risks, and meet the requirements of consumers and regulators regarding environmental protection [10]. Social sustainability refers to the need for companies to focus on social justice and social responsibility, establish good partnerships with stakeholders, and actively contribute to the betterment of the community [11]. Scholars have mainly explored the factors influencing corporate sustainability using two dimensions: green innovation drive and organizational management. First, the sustainable development of enterprises requires them to develop breakthrough green innovations on a continuous basis. Based on prior research, green innovation can be categorized into two types: green product innovation and green process innovation. In the process of implementing green innovations, enterprises can not only establish a good corporate image [12] and obtain a competitive environmental premium [13] through green product innovation but also reduce resource consumption [14] and facilitate environmental protection [15] from the source, thus improving the sustainable development advantages of enterprises. Second, attaining sustainable development requires environmental awareness to be embedded in the management of the enterprise. Specifically, executives’ dual environmental cognition (opportunistic and responsible environmental cognition) can significantly increase green technology and management innovation, and government regulatory pressure and media attention pressure also have positive moderating effects on green innovation, both of which lead to the conditions necessary for sustainable corporate development [16].

### 2.2 Review of the literature on digital transformation and enterprise sustainability

The digital transformation of enterprises constitutes a systematic endeavor, encompassing not only the adoption of digital technology within the organization but also its comprehensive influence on production and business models. In fact, the digital transformation of enterprises refers to a deep integration process between the industry and digital technology to accomplish an economic transformation. Academics have paid substantial attention to the digital transformation of enterprises. Nevertheless, the majority of the literature in this domain has concentrated on the economic implications of digital transformation, emphasizing two key aspects: the enhancement of internal efficiency and the external environmental perceptions of microenterprises. This perspective primarily examines specific information technology domains like digital finance and the internet. Liu, Shuchun, et al. found that enterprises’ implementation of digital management projects can improve their input‒output efficiency and thus their economic performance based on the "two-chemistry" integration pilot policy [17]. Wu Fei et al. conducted an investigation into the depth of corporate digital transformation using web crawling techniques. Their study revealed that corporate digital transformation notably diminishes information asymmetry, elevates optimistic market outlooks, enhances business performance, and effectively improves stock liquidity levels [18]. Another scholar examined the link between dynamic enterprise capabilities and digital transformation focusing on total data lifecycle management, and found that enterprises conduct rapid data collection and intelligent analysis with the help of data analytics platforms, facilitating the identification of potential business opportunities and environmental threats from a multidimensional perspective [19]. In addition, some scholars have conducted practical investigations of the enterprise division of labor [20] and supply chain integration [21].

The sports industry is an emerging driver in China’s national economic growth, characterized as both a green industry and a burgeoning sector. It is also a health industry and a happiness industry, thereby playing an irreplaceable role in meeting individuals’ increasing needs for a better life. Sports enterprises, as an essential part of the sports industry, significantly contribute to driving digital innovation and transformation within the sports industry while also assessing the extent of digital adoption within this sector [22]. As a result, scholars are highly interested in the digital transformation of sports corporations. The literature on this topic has highlighted two main aspects. On the one hand, at the theoretical level, Han Chaoyang et al. examined the fundamental requirements of the transformation of the meso sports industry from macro and mesolevel perspectives and concluded that the digital transformation of sports enterprises has the potential to enhance the systemic structure of sports enterprises, reshape the marketing system, improve management efficiency, and help expand the economic benefits of sports enterprises from the micro level [23]. Shen Keyin et al. combined typical cases to explore the background of the digital transformation of the sports industry in terms of the four levels of the development concept, production factors, value creation, and industrial development with the goal of investigating how digital transformation promotes the integration of resources in the sports industry; these authors have also proposed practical strategies for the digital transformation of the sports industry in China [24]. Li Yanli et al. examined the emerging infrastructure and the technological underpinnings related to the digital transformation of sports enterprises. These authors proposed specific digital transformation measures intended to increase the supply of support policies, support the development of key application areas, and foster and cultivate digital sports consumers [25]. On the other hand, at the empirical analysis level, Mou Shiminglin et al. found that digital transformation improves firms’ total factor productivity (TFP) through cost reductions and human capital optimization. Conversely, there is a time delay in the mediating impact of innovation levels on the connection between these two factors. The study’s findings offer additional empirical substantiation for promoting the high-quality development of sports firm [26]. Xu Jinfu et al., based on 26 sports enterprises listed on the A-shares market and New Third Board, concluded that the digital transformation of sporting goods manufacturing enterprises improves business performance through two paths, i.e., enhancing operational efficiency and diminishing operational expenditures. Furthermore, Digital transformation’s impact on enhancing business performance exhibits heterogeneity between market-affiliated and factor-intensive enterprises [27]. In addition, some scholars have also proposed that active innovation and change in firms are essential driving forces for accelerating firm growth from the perspective of innovative transformation and change in firms [28].

Corporate sustainability is achieved in a number of ways to generate sustainable returns and capacity expansion, and scholars have recently investigated the aspects influencing corporate sustainability from many angles. Internally, contemporary research has examined the factors that influence business sustainability in terms of green intellectual capital [29], corporate governance [30], board diversity [31], and corporate social responsibility [32]. The impacts of corporate sustainability have also been studied from the perspectives of external uncertainty [33], resource-based cities [34], green growth [35], circular economy practices [36], and air quality monitoring policies [37]. Scholars such as Xu Huaining have studied the effects of digital transformation on the sustainable development of organizations at four levels: innovation, resources, culture, and management [6]. Their analysis is based on the concept of core competitiveness shaping. Using a sample of Chinese A-share listed companies in the manufacturing industry, Chen Peng explored whether digital transformation has a positive impact on corporate sustainability and empirically found that digital transformation provides a new impetus to corporate sustainability by alleviating drawbacks such as barriers to knowledge flow, industry monopoly, outflow of skilled personnel, female board disadvantage, and the aging board problem; the study found that low production cost and low cost of goods sold, high labor productivity, and high innovativeness are important channels between digital transformation and corporate sustainability [38]. Few scholars have empirically explored the relationship between resource-based cities (SDPRC) and corporate sustainable development using a sample of listed companies in China, and the results show that the SDPRC achieves corporate sustainable development by improving corporate resource utilization efficiency and green innovation output. Heterogeneity analyses have shown that the SDPRC positively affects the sustainable performance of corporations only in growing and mature cities and more positively affects state-owned enterprises (SOEs), large-scale enterprises (LSOs), and heavy polluters (HSEs), which provides a new theoretical perspective for reforming urban planning policy in developing countries such as China [34]. Unfortunately, the topic of "digital transformation and the sustainability of sports enterprises" has not been adequately addressed in the literature. Therefore, can the digital transformation contribute to the sustainability of sports companies? Do different regions and technology firms exhibit variability in this respect? If this effect is confirmed, what is the underlying mechanism explaining the influence of digital transformation on the sustainable development of sports enterprises? Previous research has notably lacked adequate empirical investigations for a precise evaluation of the digital transformation and sustainable development of sports enterprises at the micro level. This topic requires urgent research attention.

The potential incremental contributions of this study include: (1) the innovative use of the text mining method to construct a more comprehensive indicator of the digital transformation level of enterprises based on the annual reports of Chinese sports enterprises listed on the Shanghai and Shenzhen A-shares market and New Third Board, which can provide a valuable reference for assessing the digital transformation of enterprises; (2) an approach that links digital transformation with the sustainable development of sports enterprises, analyzes the relationship between "digital transformation and sustainable development of sports enterprises," expands our understanding of the sustainable development of sports enterprises at the microlevel, and enriches our understanding of the interaction between the sustainable development and digital transformation of enterprises; and (3) a research framework based on "benchmark analysis—endogeneity test—heterogeneity test—mechanism analysis," especially at the level of the chain mediation and panel threshold effects, this approach helps unveil the ’black box’ of the mechanism that underlies the connection between digital transformation and the sustainable development of enterprises. It also furnishes systematic evidence and insights into the potential of digital transformation to foster enterprise sustainability.

## 3 Theoretical analysis and research hypotheses

### 3.1 Digital transformation’s influence on corporate sustainability

Adopting a strategy for sustainable development enables enterprises to enhance their global competitiveness and expand their reach on an international scale. In the context of the digital economy, many corporations expect to achieve high performance, high efficiency, high innovation, and low cost by starting on the path toward digital transformation to promote sustainable development [39]. Evidently, the digital transformation journey has allowed companies to surpass the one-dimensional growth model. and they have gained access to greater opportunities for growth by using digital technologies to continuously change their business logic and value proposition [40, 41]. From an intrafirm perspective, the utilization of digital technology can assist companies in extending the scope and depth of their unstructured internal data, such as the new resources generated through the introduction of digital devices and technologies complement the firm’s existing resource base, resulting in diffusion effects such as increased product output and production efficiency, which can broaden the company’s resource reservoir and enhance the effectiveness of resource allocation [42]. The application of digital technology facilitates the integration of preexisting production operations and increases the rate of information flow and utilization within the enterprise by digitizing all aspects, ranging from research and development (R&D) to operations [43]. Simultaneously, features such as the interconnection and the sharing of digital technologies complete organizational disintermediation, which aids in enhancing ineffective processes within the enterprise and leads to an upgrade of the organizational structure to a more agile and highly resilient model. [44].

Exogenous growth theory posits that the external environment in which a company operates influences its long-term sustainable growth [45]. The digital environment is an essential part of the external environment in which enterprises are located. Advanced data and information mining systems facilitate the accurate collection of vast amounts of external data, alleviate the information asymmetry between supply and demand [46], ensure the timely identification of diversified consumer needs, improve decision making, and thus help provide personalized services to customers, which ultimately benefits enterprise development [47]. Due to the high rate of growth exhibited by the digital economy and strong government support for industry digitization and digital industrialization, this scenario offers a favorable external environment for the digital transformation and enhancement of sports enterprises. It offers more development opportunities and market space for enterprise growth as a new engine for promoting sustainable growth [48]. Digital transformation is thus one of the most important ways in which companies can achieve sustainable growth while adapting to the rapidly changing market environment and technological advances.

Accordingly, we propose H1:Digital transformation significantly contributes positively to the sustainable development of corporations.

### 3.2 Analysis of the multiple impact mechanisms of resource interventions

#### 3.2.1 The chain mediating effect of “digital transformation-corporate sustainability”

The new growth theory considers firm technological innovation to be an important factor influencing TFP. In the digital era, promoting the sustainable development of enterprises must prioritize technological innovation to help develop innovative products and improve TFP [26]. Digital transformation introduces new technological tools and platforms, such as embedding cloud computing, big data analytics, and artificial intelligence into production operations and organizational processes, thereby enhancing the penetration of digital technologies in enterprises and facilitating more product innovation, organizational innovation, and business model innovation [49]. Simultaneously, technologies such as digital simulation and digital twins facilitate the mapping and reconstructing of the actual material world by mirroring constructs and simulating extreme scenarios, thereby overcoming the limitations of physical space on the R&D environment, expanding the potential scope of R&D trial and error, effectively improving the traditional innovation process, and increasing the efficiency of the transformation of innovation results [50, 51]. Endogenous growth theory reveals that the digital transformation of sports enterprises can improve TFP at the technological innovation level and the efficiency of a combination of production factors [24, 52]. Therefore, due to the application of digital transformation, enterprises must achieve sustainable development by using digital resources at all levels, promoting the synergistic innovation of various elements, continuously developing sustainable competitive capabilities that meet current needs, and cultivating long-term competitive advantages [53].

Accordingly, we propose H2: "technological innovation-total factor productivity" plays an intermediary chain role in digital transformation for sustainable enterprise development, i.e., there is a transmission path of "digital transformation → technological innovation → total factor productivity → sustainable enterprise development."

#### 3.2.2 Nonlinear spillover effects on the relationship between digital transformation and corporate sustainability

Financing limitations pose a significant challenge to companies within the context of sustainable development. In the early phases of digital transformation, companies require significant capital to make investments in technology, system updates, and human resource training to adapt to the changing market environment. However, the presence of financing limitations renders it challenging for companies to secure adequate financial backing, which constrains the digital transformation process and thus affects the company’s sustainable development [54]. However, in the middle and late stages of digital transformation, firms may gradually overcome financing constraints by increasing efficiency and developing innovation capabilities [55], and this proactive response can enable them to adapt to market demand more effectively, drive continuous value creation in a competitive market, and promote sustainable growth.

Accordingly, we propose H3 as follows:Financing limitations exhibit a nonlinear spillover impact on the association between digital transformation and corporate sustainability.

As digital transformation continues to evolve, the distinctive online and intelligent attributes of digital technology can mitigate information asymmetry among all stakeholders in various transactions and curtail expenses incurred by enterprises across various stages [56]. Specifically, introducing digital technologies and automated systems can allow companies to control costs more effectively by overcoming temporal and spatial barriers and analyzing consumer preferences more accurately at zero cost [57]. Second, to improve internal process collaboration and external upstream and downstream collaboration and to reduce communication costs through interconnection at the information exchange level [27], with the help of blockchain and other technologies, sports companies can use the "shared" management concept to improve instantaneous decision-making responses across departments and levels and reduce management and production costs. However, suppose that a corporation excessively pursues the goal of reducing operating costs. This situation may lead to problems such as insufficient human resources and reduced product quality, adversely affecting the company’s sustainable development.

Accordingly, we propose H4 as follows: Operational expenses exhibit a nonlinear spillover impact on the association between digital transformation and corporate sustainability.

As the cornerstone of digital transformation, operational efficiency has the potential to enhance the quality and effectiveness of enterprises at various levels, serving as the catalyst for enterprise development. On one hand, the implementation of digital systems, such as Enterprise Resource Planning (ERP) systems and Customer Relationship Management (CRM) systems, fosters the automation and optimization of internal business processes and improvements in work efficiency [58]. Furthermore, digital transformation enhances the information mining capabilities of enterprises, enabling precise marketing and the enhancement of product market share and marketing efficiency. In addition, in the context of digital transformation, the three aspects of operational digitization, decision flexibility, and information transparency help visualize the entire supply chain and improve the efficiency of organizational operations [59]. On the other hand, an intelligent and efficient supply chain management system allows companies to manage and optimize the use of resources more effectively, reduce waste and energy consumption, and significantly optimize resource efficiency and environmental sustainability [60]. Therefore, under the combined effect of multiple factors, digital transformation improves operational efficiency at all levels of the enterprise value chain, creates more profits and competitive advantages, and drives sustainable development.

Accordingly, we propose H5 as follows: Operational efficiency exhibits a nonlinear spillover impact on the association between digital transformation and corporate sustainability.

The theoretical model employed in this study was constructed by synthesizing the relational arguments and hypotheses discussed above to define the relevant variables. Fig 1 of S1 File.

## 4 Study design

### 4.1 Data source and sample selection

Sports companies listed (quoted) on the Shanghai and Shenzhen A-shares market and the New Third Board in China from 2012 to 2021 were selected as the research sample, and the annual financial data reported by these companies were used for the empirical analysis. The following principles were followed in the sample selection.

(1) Based on the classification of the industry to which the corporation belongs and an analysis of its primary business, etc., the fact that the selected corporation conforms to the sports industry according to the Statistical Classification of Sports Industry (2019) was verified; (2) sports corporations listed (quoted) after December 31, 2021, were excluded; (3) ST, *ST, PT, and other abnormal business enterprises were excluded; and (4) some corporations with missing and abnormal data were excluded. After the screening, 48 sample enterprises were obtained, including 21 enterprises listed on the Shanghai and Shenzhen A-shares market and 27 corporations on the New Third Board. Finally, unbalanced panel data consisting of 297 annual corporation observations were obtained. The data used in this study were drawn mainly from the WIND database, the Guotaian CSMAR database, China Financial Information Network, the National Small and Medium Enterprise Share Transfer System, the Juchao Information Network, the City Statistical Yearbook, etc.

### 4.2 Empirical model

The empirical model presented below is formulated to explore the direct impact of digital transformation on corporate sustainability:

$${\text{Cs}}_{i,y}=\alpha +\beta {Dig}_{i,y}+\delta Controls+\varepsilon i+\phi y+\lambda_{i,y}$$ (1)

In eq (1), *CS*_*i*,*y*_ is the explanatory variable indicating the sustainability of corporation i in period y; *Dig*_i,y_ is the core explanatory variable indicating the level of digital transformation exhibited by corporation i in period y; *Controls* is a set of control variables; *εi* band *ϕ*y indicate fixed effects at the corporation and time levels, respectively, and *λ*_*i*,*y*_ indicates the random disturbance term.

### 4.3 Explained variables

Corporate sustainability (Cs) refers to an enterprise’s capacity to attain its business objectives, maintain its market standing, and ensure its long-term viability. This involves sustaining its competitive edge in its current domains while also adapting to the evolving business landscape of the future, ultimately ensuring profitability and consistent growth over an extended period. The measures used most recently for sustainability include the Higgins Sustainable Growth Model and the Van Horne Sustainable Growth Model, given that the Higgins Sustainable Growth Model does not consider the dynamic growth of corporations. Therefore, drawing on the research results reported by Yang Dongxu [9], we construct corporate sustainability indicators in accordance with the Van Horn Sustainable Growth Model to measure the sustainability of listed sports companies.

### 4.4 Core explanatory variables

Digital transformation level (Dig). Drawing on the methods used to measure the level of digital transformation in economics and management, the frequency of digitization-related keywords in the annual reports of sample enterprises was counted using the text mining method; in addition, to make the measurement results more convincing, we quantified the extent of digital transformation in corporations by calculating the ratio of keyword frequencies in the annual reports of the sample companies to the total keyword frequencies in the annual reports of all companies within that specific year [27]. In general, the higher the word frequency of the target keyword is, the more attention and resources the company devotes to the keyword and the deeper is the development [18].

The assessment of an enterprise’s digital transformation level comprises two components. In the first part, a digital keyword thesaurus for sports corporations is constructed. Initially, we construct a keyword summary table for the sample enterprises, encompassing the five dimensions of big data technology, digital technology application, artificial intelligence technology, cloud computing technology, and blockchain technology. This table is developed by referencing the characteristics of digital transformation within sports corporations to establish a comprehensive keyword thesaurus. On this basis, the keyword thesaurus is expanded, and the expanded keywords are adjusted using the Delphi method to develop the final thesaurus. The second part—keyword word frequency statistics—uses the word splitting function in Python and Jieba software. The annual reports of corporations are processed by word splitting, and the frequency with which keywords occur is counted to obtain corporations’ digital transformation levels.

### 4.5 Intermediate variables

R&D investment is the starting point for corporate technological innovation (Rd), and we choose to measure the corporation’s level of technological innovation in terms of the proportion of R&D investments to operating revenue [27]. TFP is measured using the control function method (LP method) and draws on the calculation method proposed by Mou Shiming Lin et al. [26].

### 4.6 Threshold variables

Financing constraints (Fc). We utilize the SA index as a metric for quantifying the extent of financing constraints [61]. The specific formula for the SA index is

$$SA=-0.377\times \text{Size}+0.043\times {\text{Size}}^{\text{2}}-0.04\times Age$$ (2)

In eq (2), *Size* denotes the natural logarithm of the corporation’s total assets at the end of the period, and *Age* denotes the number of years since the corporation’s establishment. The higher the absolute value of the *SA* index is, the higher is the degree of financing constraints on the corporation.

The total operating cost expense ratio measures operating costs (Cost). The higher the total operating cost-expense ratio is, the higher is the input costs of the corporation. The total asset turnover ratio measures operational efficiency (Eff); the higher the total asset turnover ratio is, the more opportunities for asset utilization the corporation has and the higher is its operational efficiency [27].

### 4.7 Control variables

To reduce the interference of multiple factors in the estimation of causal effects in the multiple regression analysis, the following control variables are introduced at the corporation level based on the results of previous research: corporate size (Size), gearing ratio (Lev), corporate growth (Growth), board size (Dp), supervisory board size (Gp), unrestricted shares outstanding (Lt), and capital intensity (Cap); in addition, the analysis controls for year (Year) and individual firm (Id). Table 1 shows the specific metrics used for each variable.

**Table 1:** Descriptions of the study variables.

Variable Category	Variable Name	Variable Symbol	Variable Definition and Description
Explained variables	Corporate sustainability	Cs	Corporate Sustainability = Net sales margin × Earnings retention rate × (1+Equity ratio)/[1/Net sales margin × Earnings retention rate × (1+Equity ratio)]
Explanatory variables	Digital transformation level	Dig	Frequency of digitized keyword terms in corporate annual reports/Total number of digitized keyword terms in the annual report of the entire sample of corporations in the current year
Intermediate variables	Technological innovation	Rd	R&D investment/Operating income (taking the logarithm)
	Total factor productivity	TFP	Total factor productivity of corporations calculated using the LP method
Threshold variables	Financing constraints	Fc	Calculated SA index
	Operational efficiency	Eff	Operating income/Average total assets
	Operating costs	Cost	Total operating costs/Total operating revenue
Control variables	Corporate size	Size	Natural logarithm of total assets at the end of the reporting period
	Gearing ratio	Lev	Total liabilities/Total assets
	Corporate growth	Growth	(Operating income for the current year-Operating income for the prior year)/Operating income for the prior year
	Supervisory board size	Gp	Number of supervisory committee
	Board size	Dp	Number of board of directors
	Unrestricted shares outstanding	Lt	Size of unrestricted shares outstanding (taking the logarithm)
	Capital Intensity	Cap	Fixed assets/Number of employees

## 5 Empirical results and analysis

### 5.1 Descriptive statistics and multicollinearity test

Table 2 presents descriptive statistics and results of covariance tests for each variable. It reveals that the mean value of corporate sustainability is 0.1321, with a standard deviation of 0.5828. The mean value of digital transformation is 0.0325 with a standard deviation of 0.0775, indicating some degree of variability and a high degree of dispersion among the samples. All of these variables have more room for improvement in light of those associated with corporations in other fields. According to Accenture statistics, the digital gap between digital transformation leaders and other corporations gradually widened between 2018 and 2021, especially regarding strategy development capabilities, highlighting a snowball effect [62]. Therefore, corporations should actively cooperate and engage in joint construction, consciously strengthen their exchanges with high-quality industrial internet companies in terms of industrial software, automation, and other digital-related technologies, focus on establishing an excellent industrial development environment, and jointly promote the coordinated development of the digital transformation and sustainable development of corporations. The highest Variance Inflation Factor (VIF) observed among all variables is 3.59, which is below the threshold of 5, indicating the absence of multicollinearity issues.

**Table 2:** Descriptive statistics of variables and VIF.

Variables	Mean	Sd	Min	Max	VIF	1/VIF
Cs	0.1321	0.5828	-1.7135	3.0607	—	—
Dig	0.0325	0.0775	0.0000	0.8571	1.41	0.708902
Rd	1.2987	0.8477	0.0000	5.3533	1.57	0.635207
TFP	5.7329	0.4751	2.6478	7.3270	2.42	0.412920
Fc	-3.7435	0.2350	-4.4561	-3.1651	1.71	0.586286
Cost	1.2988	6.4844	0.2128	97.4393	1.62	0.618322
Eff	0.8413	0.5382	0.0005	3.0831	1.67	0.598061
logSize	19.5976	1.9457	14.7009	23.0873	3.57	0.280348
Lev	43.2444	21.8663	0.8300	120.8900	1.26	0.790913
Growth	0.9542	8.1467	-0.9956	128.9411	1.20	0.832207
Gp	3.0842	0.4064	2.0000	5.0000	1.15	0.866117
Dp	6.7643	1.8302	4.0000	11.0000	2.49	0.401213
logLt	3.7207	1.5549	0.0000	15.6969	1.32	0.759807
Cap	182,000	481,000	569.8299	7240000	1.26	0.792884
Mean VIF	—	—	—	—	3.59	

### 5.2 Baseline regression analysis

Using Stata 15.3 software, a fixed effects model was used for the analysis following the Hausman test.

Table 3 shows the regression results of Model (1) and that the digital transformation on corporate sustainability has an impact coefficient of 0.1225 when no control variables are added. For Model (2), after adding the control variables for both corporate and year-fixed effects, the coefficient of the impact of digital transformation on corporate sustainability is 0.3627. This model successfully passes the significance test at the 1% level, demonstrating a significant and positive association between digital transformation and corporate sustainability in a general context. H1 is thus confirmed.

**Table 3:** Baseline regression results of the impact of digital transformation on corporate sustainability.

Variable	Models (1)	Models (2)
Dig	0.1225	0.3627***
	(1.56)	(2.84)
logSize		0.3370***
		(5.19)
Lev		-0.0111***
		(-7.53)
Growth		0.0028**
		(2.03)
Gp		-0.0376
		(-0.87)
Dp		-0.0528
		(-1.65)
logLt		-0.0223
		(-0.98)
Cap		0.0000
		(0.89)
_cons	0.0627*	-5.4582***
	(1.77)	(-4.53)
Year/Id	No	Yes
N	297	297
r2	0.0325	0.3973

**Note:** Test t in parentheses. ***, **, * indicate significance at the 1%, 5%, and 10% levels, respectively; the same holds true below.

### 5.3 Robustness tests and discussion of endogeneity

#### 5.3.1 Robustness test

To ensure the reliability of the research results, the study examines the robustness of its findings regarding the influence of digital transformation on sustainable corporate development. First, the sample size is reduced, which is accomplished by randomly eliminating ten corporations each that are listed on the A-shares market and New Third Board; the regression results for Model (3) are shown in Table 4. Second, the fixed effects of the benchmark model are set to a higher-order form, including time and industry dimension interactions, while controlling for corporate-level fixed effects and changing the original model settings for the robustness test; the regression results for Model (4) are shown in Table 4. From Models (3) and (4), the estimated coefficients are 0.4829 and 0.3929, respectively, which are significant at the 1% level. The core conclusion is that "digital transformation helps enhance the sustainable development of corporations," these results align with those obtained from the benchmark regression, underscoring the reliability and validity of the study’s findings.

**Table 4:** Robustness tests and estimation results of the instrumental variables.

Variable	Robustness tests		Instrumental variable estimation	
	Reduced sample size	Higher-order interaction fixed effects	Number of fixed telephones × employees	Number of post offices × employees
	Models (3)	Models (4)	Models (5)	Models (6)
Dig	0.4829***	0.3929***	2.6900*	2.5927*
	(3.64)	(3.06)	(1.81)	(1.93)
IV1			2.8140*	
			1.77	
First Stage: F			14.8***	
	R-squared			0.0443	
IV2				2.1037**
				2.47
First Stage: F				16.94***
R-squared				0.0474
Control variables	Yes	Yes	Yes	Yes
Year/Id	Yes	Yes	Yes	Yes
_cons	-6.4749***	-5.2859***	-3.4012***	-3.3969***
	(-2.91)	(-4.31)	(-4.18)	(-4.28)
N	192	297	297	297

#### 5.3.2 Endogeneity discussion: Instrumental variables approach

Although using higher-order interaction fixed effects helps mitigate the endogeneity problem, in this study, the sustainable development of corporations cannot be isolated from corporate digital transformation. In addition, the corporation’s degree of digital transformation must be linked to its long-term development. Thus, there may be indications of reverse causality between the level of digital transformation within the corporation and its sustainable development. On the other hand, many factors affect corporate sustainability, and the control variables included in the current study make it difficult to avoid the creation of omitted variables. Therefore, to capture more accurately the relationship between digital transformation and corporate sustainability, we identify the net effect of digital transformation on corporate sustainability by mitigating possible endogeneity issues through an instrumental variables approach.

Specifically, we draw on the studies of Shuo-Yi Peng [63] and Qun-Hui Huang et al. [64] and use the number of fixed post offices and telephone calls in 1984 in the city in which the corporation is located as instrumental variables. These instrumental variables meet both the relevance and exclusivity constraints. On the one hand, digital transformation is premised on the rapid development of the regional internet, which serves as a continuation of traditional communication methods such as telephones; thus, it is likely that cities with historically high fixed-line penetration rates also have high internet penetration rates. In addition, before the widespread adoption of the fixed-line telephone, individuals communicated mainly through the post office system. The distribution of post offices impacts the distribution of fixed-line telephones and, hence, the initial access to the internet. This situation thus satisfies the relevance criterion. On the other hand, historical post offices, telephones, and other conventional forms of communication have little impact on the long-term growth of modern sports corporations. This situation thus satisfies the exclusivity criterion. It’s important to note that we employ unbalanced panel data, and the chosen instrumental variables are in a cross-sectional format, making them unsuitable for direct application in the econometric analysis of panel data. Accordingly, a time-varying variable is incorporated to create the panel instrumental variable, utilizing the methodology outlined by Nunn and Qian [65]. We construct interaction terms for the number of post offices per million people and the number of landlines per 100 people in 1984 in the city in which the firm is located using the number of people employed in computer services and software (time-dependent) in each city in the previous year as two instrumental variables for the digital transformation of the corporation. Table 4 present the regression results of the instrumental variables of Models (5) and (6). After accounting for the potential endogeneity between digital transformation and corporate sustainability, the coefficient associated with corporate digital transformation remains positive and statistically significant. This suggests that the extent of corporate digital transformation can indeed make a substantial contribution to corporate sustainability, aligning perfectly with the findings of prior research.

### 5.4 Heterogeneity analysis

The preceding benchmark regression examines the impact of digital transformation on the sustainability of the entire sample of corporations. Notably, variability in terms of the region in which each company is located and corporate size and type of business may lead to asymmetric effects of digital transformation on sustainable corporate development. Therefore, employing group regression analysis to delve deeper into the influence of digital transformation on corporate sustainability, considering distinct regional and technological characteristics, can aid companies in formulating tailored digital transformation strategies.

#### 5.4.1 Regional heterogeneity test

The results of Models (7) and (8) in Table 5 show that digital transformation has a significant positive effect on the sustainable development of corporations in the eastern region of China. In contrast, the effect is nonsignificant in the central and western regions. This finding shows that the positive effect of digital transformation on the sustainable development of corporations is more substantial in eastern China. According to Chandler [66], a corporation’s sustainability is the result of an entrepreneur’s continuous response to new technologies and new market demands. Since the COVID-19 pandemic, global digital transformation spending has continued to grow rapidly. Overall, China, the United States, and Europe form a tripolar pattern for the development of the global digital economy, in which context China’s digital economy has achieved leapfrog development and is second only to that of the United States in terms of scale [1]. In particular, the eastern region of China, which is closer to the international market and has a good business environment, has natural advantages in areas such as access to resources and market share. Furthermore, local governments have guided banks and other financial institutions to provide financial support for corporations to promote the digitalization, informatization, and intellectualization of enterprises, which can directly improve the scale and degree of financing of corporations and solve the problems associated with "difficult" and "expensive" financing; thus, China’s eastern region has a "first mover advantage" over its central and western regions [67]. Therefore, in a fiercely competitive environment, sports corporations in eastern China must keep pace with the development of the digital economy and stimulate the potential for digital transformation and upgrading if they want to align themselves with new technologies and markets in developed countries [68]. In general, the willingness of sports corporations in the eastern region of China to promote digital transformation and the urgency of this task are more vital than in the central and western regions of China and thus has a stronger effect on sustainable development.

**Table 5:** Heterogeneity test based on regional attributes and technical attributes.

Variable	Eastern region (7)	Midwestern region (8)	Nonhigh-tech corporation (9)	High-tech corporation (10)
Dig	0.2698**	2.3292	1.9946	0.3588***
	(2.16)	(1.22)	(0.92)	(2.96)
logSize	0.3048***	0.4307*	0.2397***	0.3120***
	(4.45)	(2.18)	(3.61)	(3.50)
Lev	-0.0079***	-0.0165***	-0.0147***	-0.0082***
	(-5.05)	(-3.84)	(-6.52)	(-4.12)
Growth	0.0014	0.0308	-0.0382	0.0020
	(0.90)	(1.44)	(-0.99)	(1.04)
Gp	-0.0276	-0.0718	-0.3123**	-0.0530
	(-0.71)	(-0.81)	(-3.01)	(-0.85)
Dp	-0.0364	-0.1341	0.0733	-0.0493
	(-1.01)	(-1.13)	(1.38)	(-1.24)
logLt	-0.0734*	0.0104	-0.0008	-0.0517
	(-1.81)	(0.85)	(-0.05)	(-1.45)
Cap	0.0000	-0.0000	0.0000	0.0000
	(0.52)	(-0.96)	(0.09)	(0.92)
_cons	-5.0139***	-6.0727*	-3.4511**	-5.0172***
	(-3.96)	(-2.04)	(-2.59)	(-3.09)
Year/Id	Yes	Yes	Yes	Yes
N	239	58	57	240
r2	0.3191	0.7125	0.8226	0.2392

#### 5.4.2 Technical heterogeneity test

For corporations, digital transformation is a long-term strategic endeavor. Corporations’ strong competitive edge and higher levels of innovation ability and economic efficiency have greater impacts on their long-term development. Table 5 presents the outcomes of Models (9) and (10), demonstrating a noteworthy positive effect of digital transformation on the sustainable development of high-tech corporations. However, simultaneously, digital transformation fails to impact nonhigh-tech corporations significantly, indicating that digital transformation by high-tech corporations plays a more significant role in the sustainable development of corporations. The reason for this finding is that, on the one hand, high-tech enterprises are economic entities that are technology- and knowledge-intensive, and the critical focus of production and operation in this context is on scientific and technological innovation; enterprises themselves are closer to the technology and frontier aspects of their fields and have more robust demand for updating and iteration, which naturally increases their willingness to promote digital transformation [69, 70]. Second, high-tech corporations can effectively satisfy the creative technical conditions and talent support required for digital transformation. They may deeply integrate digital transformation into their organizational structure and manufacturing processes. In addition to the high level of importance they attribute to digital transformation and corporate management’s sensitivity and insights into advanced technologies [71], in this context, the benefits and opportunities of digital transformation were previously fully exploited to achieve market expansion, business model innovation, and operational efficiency improvements, and the sustainable development dividend offered by the current digital transformation may have been realized to some extent [18]. From a comprehensive perspective, the objective basis and active willingness of high-tech corporations to promote digital transformation render their attempts to promote the digital transformation process more effective than that of nonhigh-tech corporations. High-tech corporations are also more capable of exploiting the enhancement effect of digital transformation on sustainable corporate development.

## 6 Analysis of the chain mediating effects

The findings of the examination for chain mediating effects are displayed in Table 6. Based on Model (11), the influence of digital transformation on corporate sustainability is statistically significant and positive. In accordance with Model (12), the impact of digital transformation on technological innovation is significantly positive. This result further underscores the capacity of digital transformation to enhance technological innovation within sports corporations. For Model (13), the regression coefficients of digital transformation and technological innovation on TFP are significantly positive and significantly negative, respectively, indicating that digital transformation is conducive to improving the TFP of sports corporations, while enhancements to corporation’s technological innovation are detrimental to their TFP. In Model (14), the regression coefficients for digital transformation and the TFP of corporations are notably positive and statistically significant. However, the coefficient of technological innovation does not have a significant effect, indicating that no chain mediating effect exists. H2 is thus not confirmed. The reason for this finding is that the digital transformation of Chinese sports corporations is still in its infancy. High investments in technological innovation and the lag in benefits have primarily prevented technological innovation from having immediate effects on the TFP and sustainable development of corporations [27]. On the other hand, due to the imitation effect of core technology, investments in technological innovation made by sports corporations fail to play a substantial role in promoting enterprise development. In contrast, these investments may lead to tremendous sunk costs. Therefore, the promotional effect of technological innovation on corporate development has yet to be revealed [26].

**Table 6:** Results regarding the chain mediating effect of "digital transformation-corporate sustainability".

Variable	Cs	Rd	TFP	Cs
	Models (11)	Models (12)	Models (13)	Models (14)
Dig	0.3627***	1.2082***	0.2879**	0.3314**
	(2.84)	(4.70)	(2.02)	(2.67)
Rd			-0.1909***	0.0184
			(-8.60)	(0.38)
TFP				0.1580***
				(3.25)
_cons	-5.4582***	1.2019	0.8738	-5.5822***
	(-4.53)	(0.53)	(0.50)	(-4.67)
Year/Id	Yes	Yes	Yes	Yes
N	297	297	297	297
r2	0.3973	0.2088	0.2824	0.4127

## 7 Threshold effect analysis

### 7.1 Constructing a panel threshold regression model

Based on the aforementioned theoretical analysis, the relationship between digital transformation and corporate sustainability may be characterized differently when the threshold variables are in different intervals, i.e., there may be nonlinear spillover effects among the variables. Therefore, we employ Hansen’s (1999) panel threshold regression model to examine the potential non-linear relationship among the variables, and the following panel threshold model is developed [72]:

$$\begin{array}{ll} {\text{Cs}}_{i,y} & =\varphi_{0}+\varphi_{1}{\text{Cs}}_{i,y}\times I\left({Adj}_{i,y}\leq \theta_{1}\right)+\varphi_{2}{\text{Cs}}_{i,y}\times I\left({\theta_{1}<Adj}_{i,y}\leq \theta_{2}\right)\\ & {+\varphi }_{3}{\text{Cs}}_{i,y}\times I\left({\theta_{2}<Adj}_{i,y}\leq \theta_{3}\right){+\varphi }_{4}{\text{Cs}}_{i,y}\times I\left({Adj}_{i,y}>\theta_{3}\right)\\ & +\eta {Controls}_{i,y}+\omega_{i,y} \end{array}$$ (3)

In eq (3), *ADJ*_*i*,*y*_ represents threshold variables such as financing constraints, running costs, and operating efficiency; *θ* represents the threshold value, and *I* (·) represents the indicator function, assuming a value of 1 when the condition inside the parentheses is met and 0 otherwise. *θ*_1_ = *θ*_2_ = *θ*_3_ when the threshold variable has a single threshold effect, *θ*_1_ ≠ *θ*_2_ = *θ*_3_ when the threshold variable has a double threshold impact, *θ*_1_ ≠ *θ*_2_ ≠ *θ*_3_ when the threshold variable has a triple threshold effect, *Controls*_*i*,*y*_ is the control variable, and *ω*_*i*,*y*_ is the random disturbance term. The panel threshold model shown above is then empirically tested.

### 7.2 Test of the existence of the threshold effect and the estimation results

The existence of the threshold effect was tested using a repeated sampling approach featuring 300 resamples and Hansen’s (1999) "bootstrap" method. The test outcomes, as displayed in Table 7, indicate that the financing constraints and operating cost threshold variables pass the single threshold effect test, operating efficiency passes the double threshold test, and the F-statistic is significant at least at the 10% level, thus, demonstrating threshold effects concerning financing constraints, operating costs, and operational efficiency on the influence of digital transformation on enterprises’ sustainable development. To demonstrate the determination of the threshold value and the establishment of its confidence interval, we generate a graph depicting the threshold likelihood ratio function, and the results are shown in Figs 2 and 3 of S1 File.

**Table 7:** Results of the test of the existence of the threshold effect.

Threshold variables	Number of thresholds	F	P	10% threshold	5% threshold	1% threshold	Threshold	95% confidence interval	
								Lower limit	Upper limit
Fc	Single threshold	10.49	0.077	9.961	12.812	17.362	-3.5637	-3.5982	-3.5541
Cost	Single threshold	20.80	0.037	15.211	17.892	25.858	0.7847	0.7822	0.7884
Eff	Single threshold	19.24	0.007	13.280	15.136	18.911	0.8614	0.7940	0.8616
	Double threshold	11.85	0.087	11.572	13.093	17.221	1.0670	1.0392	1.0686

Table 7’s threshold values are shown in Figs 2 to 4 (Figs 2 to 4 of S1 File). In these figures, the horizontal axis denotes the threshold, the vertical axis is labeled as LR, representing the likelihood ratio statistic, and the dashed line represents the 95% significance reference line. These figures reveal that the single threshold estimate for the presence of financing constraints is -3.5637, the single threshold estimate for the presence of operating costs is 0.7847, and the double threshold estimates for operating efficiency are 0.8614 and 1.0670, respectively. These thresholds are all much higher than the 0.05 level, indicating that the aforementioned points are correct and valid.

### 7.3 Threshold regression results

The panel threshold regression results are shown in Table 8. In Model (15), when the financing constraint is less than -3.5637, digital transformation has a negative impact on corporate sustainability. However, the impact coefficient fails the significance test, thus indicating that an excessive financing constraint may inhibit the effect of digital transformation on promoting corporate sustainability. When the financing constraint is greater than the threshold, digital transformation has a significant positive impact on corporate sustainability. This finding suggests that the spillover effect of digital transformation and corporate sustainability has a positive and nonlinear relationship with increasing "marginal effects" and that while financing constraints may harm sustainability in the early stages of change, corporations can overcome financing constraints and promote sustainable development by implementing proactive measures in the middle and late stages of change. H3 is thus confirmed.

**Table 8:** Results of parameter estimation of the panel threshold model.

Variable	Model (15)		Model (16)		Model (17)
Dig(Fc≤-3.5637)	-0.4600	Dig(Cost≤0.7847)	0.4746*	Dig(Eff≤0.8614)	-1.6359*
	(-0.52)		(1.77)		(-1.82)
Dig(Fc>-3.5637)	0.4768*	Dig(Cost>0.7847)	0.2812	Dig(0.8614<Eff≤1.0670)	0.3406
	(1.81)		(0.27)		(1.31)
				Dig(Eff>1.0670)	2.7645***
					(2.77)
Control variables	Yes		Yes		Yes
Year/Id	Yes		Yes		Yes
N	297	N	297	N	297
r2	0.3812	r2	0.3782	r2	0.4104

Based on Model (16), it is observed that the positive impact of digital transformation on corporate sustainability diminishes as operating costs increase. When operating costs exceed the threshold value of 0.7847, digital transformation has no significant impact on enterprise sustainability, indicating that these costs have a negative and "marginal effect" on the relationship between digital transformation and enterprise sustainability, thus indicating a decreasing nonlinear relationship. This finding supports the claim that operational costs are not nonvalue-added assets with a positive influence but, rather, can be negatively impacted by disproportionate or excessive increase in operating costs, which become "liabilities" [73]. In other words, higher operating costs are not better. This situation occurs mainly as a result of financial pressure. Digital transformation requires substantial investments, and an increase in operating costs can have a negative impact on the financial situation of corporations and cause them to face financial strain. Thus, their ability to invest in and allocate resources to digital transformation becomes limited, leading to a relaxation or interruption in the transformation process and limitations to the impact of digital transformation on the sustainable development of corporations and the role of digital transformation in driving sustainable growth. H4 is thus confirmed [38].

The outcomes of Model (17) reveal that the impact of digital transformation on corporate sustainability varies significantly across different degrees of operational efficiency. When operational efficiency falls below the first threshold of 0.8614, the impact of digital transformation on corporate sustainability is negative and significant. When operational efficiency is between the first and second thresholds, digital transformation has a marginally positive but nonsignificant impact on corporate sustainability. Finally, when operational efficiency exceeds the second threshold of 1.0670, digital transformation exhibits a notable positive influence on corporate sustainability The reason for this situation could be that when operational efficiency is low, the corporation requires additional resources to facilitate the implementation of digital transformation, resulting in an inability to implement digital transformation successfully and limiting the corporation’s potential for sustainable growth. When operational efficiency improves, the efficiency spillover and value diffusion from sports corporations that expand the scale of digital transformation improve, enhancing long-term growth [38].

## 8 Conclusions and recommendations

### 8.1 Conclusion

This study takes 48 sports enterprises on the A-share and New Third Board from 2012 to 2021 as research samples and examines the relationships and mechanisms of action between them from a multidimensional perspective through text mining and model building. The following conclusions are reached.

(1) Digital transformation makes a significant positive contribution to the sustainable development of sports corporations, and This conclusion remains consistent even after subjecting the results to rigorous robustness tests, such as reducing the sample size and introducing instrumental variables. (2) The heterogeneity study showed that among the different regional attributes, the incentive utility of digital transformation for sustainable development is obvious in Eastern China and in high-tech enterprises. (3) In terms of the transmission mechanism, digital transformation can promote enterprise technological innovation, but technological innovation has a negative impact on total factor productivity, and the impact on sustainable development is not significant; moreover, the chain intermediary effect of "digital transformation→technological innovation→total factor productivity→sustainable development of enterprises" has not been verified. (4) Financing constraints and operational costs exhibit a singular threshold effect on the correlation between digital transformation and corporate sustainability, and operational efficiency has a double threshold effect on the relationship between digital transformation and corporate sustainability. In this context, financing constraints exhibit a positive and increasing "marginal effect" nonlinear relationship, and operating costs exhibit a negative and decreasing "marginal effect" nonlinear relationship. Operating efficiency has a monotonically growing nonlinear positive impact on the relationship between the two factors.

### 8.2 Recommendations

1. Serve a role of policy support and create a favorable business environment. First, policy support should address the difficulties in the digital transformation of sports enterprises and target the policy. The foundation base of solid digital transformation further increases policy support for enterprise digitization, strengthens financial support for the digital transformation of sports enterprises, and alleviates the difficulties of enterprise financing [74]. Second, the government should actively create a digital intelligent service platform for the sports industry, organize relevant training on a regular basis, create a good ecological environment for the digital transformation of enterprises, fully stimulate the vitality of enterprises, constantly improve and strengthen the approval and application of supervision and management mechanisms for relevant support funds, etc., and better guide and incentivize financial support for the digital transformation of sporting goods manufacturing enterprises. In addition, enterprise development sustainability should be promoted [27].
2. Explore the path of digital transformation in accordance with the principle of "differentiation by enterprise". In the era of the digital economy, the transformation focus and realization path of different regions and different technical sports enterprises are bound to differ; therefore, enterprises should actively introduce digital technology and improve digital awareness according to their own actual situation, create a suitable digital transformation strategy, gradually integrate digital technology and concepts into all aspects of production, operation and management, and avoid blindly following the trend. Moreover, in the process of digital transformation, sports enterprises need to better cope with the problems caused by strategic mismatch, minimize the sunk cost effect, and promote high-quality sustainable development [8, 38].
3. Emphasize digital transformation and improve management systems. Enterprise management should recognize the positive effects of digital transformation on sustainable development, pay attention to the digital transformability of the enterprise, and actively adopt a digital transformation strategy that encourages employees to actively use digital tools and technologies, improves operational efficiency, and encourages technological innovation. In addition, enterprise managers should establish a perfect resource utilization and supply chain system to effectively guide the digital transformation of the enterprise and provide a source of vitality for its sustainable development [36–38].

## 9 Limitations of the study

This study’s limitations and some suggestions are discussed as follows: First, a comprehensive evaluation system should be established. Due to data limitations, this study employs corporate economic sustainability measures as explanatory variables to assess the sustainability of publicly traded sports organizations. However, enterprise sustainable development is the result of numerous factors, and in the future, we can introduce social and environmental sustainability indicators to construct a multifaceted enterprise sustainable development indicator system, allowing us to more thoroughly analyze the impact of digital transformation on enterprise sustainable development. Second, this study enriches the understanding of impact mechanisms. The impact of digital transformation on corporate sustainability is multifaceted, and this study investigates only chain-mediated and threshold effects, ignoring the mediating and moderating effects of internal and external environmental factors such as financing constraints, risk-taking levels, degrees of marketization, and regulatory pressure. Third, heterogeneity was analyzed in multiple dimensions. The scope of this analysis is limited to the level of technological and regional heterogeneity; in the future, it can be extended and expanded to include life cycle heterogeneity, firm size heterogeneity, industry heterogeneity, and heterogeneity in property rights [34]. Fourth, the sample size should be expanded. Restricted by the availability of data and consistency of statistical caliber, the sample of this study includes only A-share and New Third Board sports enterprise data, and future studies can include small and microenterprises, as well as Hong Kong, Macao, Taiwan and other sports enterprises.

## Supporting information

S1 Dataset The raw data. (XLS)

S1 File (DOC)
