# Supplementary material for: An empirical assessment of the influence of digital transformation on sports corporate sustainability
Source: PLoS One. 2024 Apr 18;19(4):e0297659. doi: 10.1371/journal.pone.0297659 (PMC11025921; doi:10.1371/journal.pone.0297659)
Supplement: S1 File — (DOC) [file pone.0297659.s002.doc]

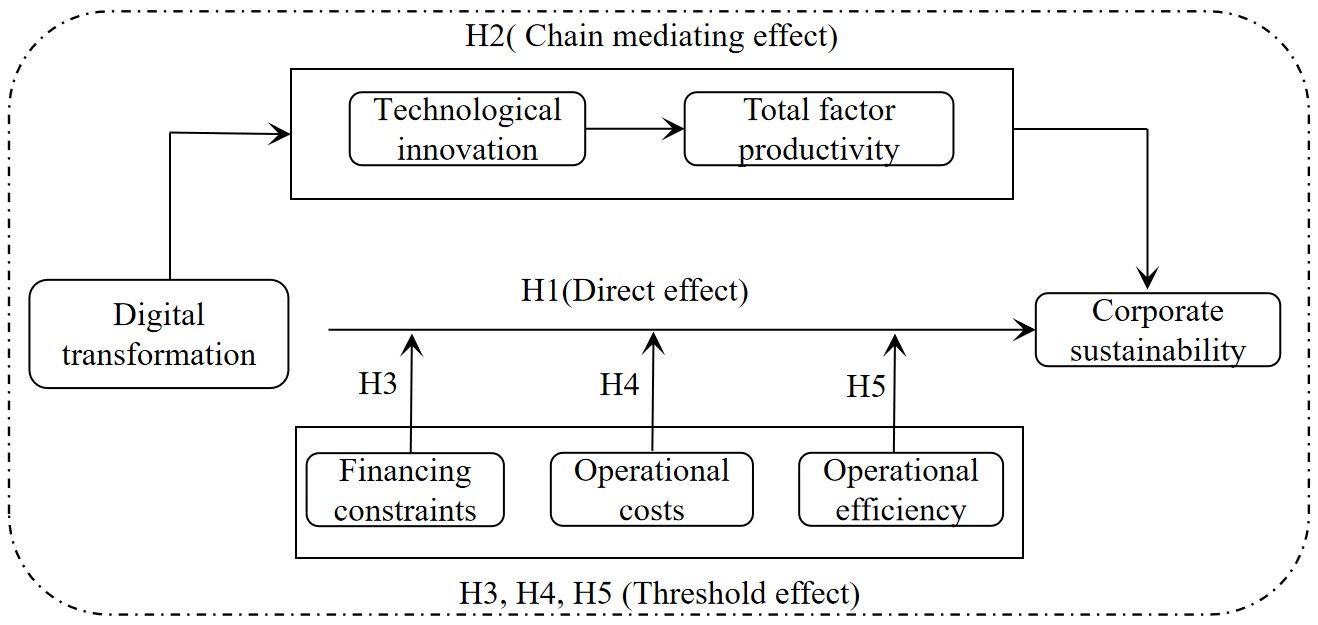


**Fig 1. Theoretical model**


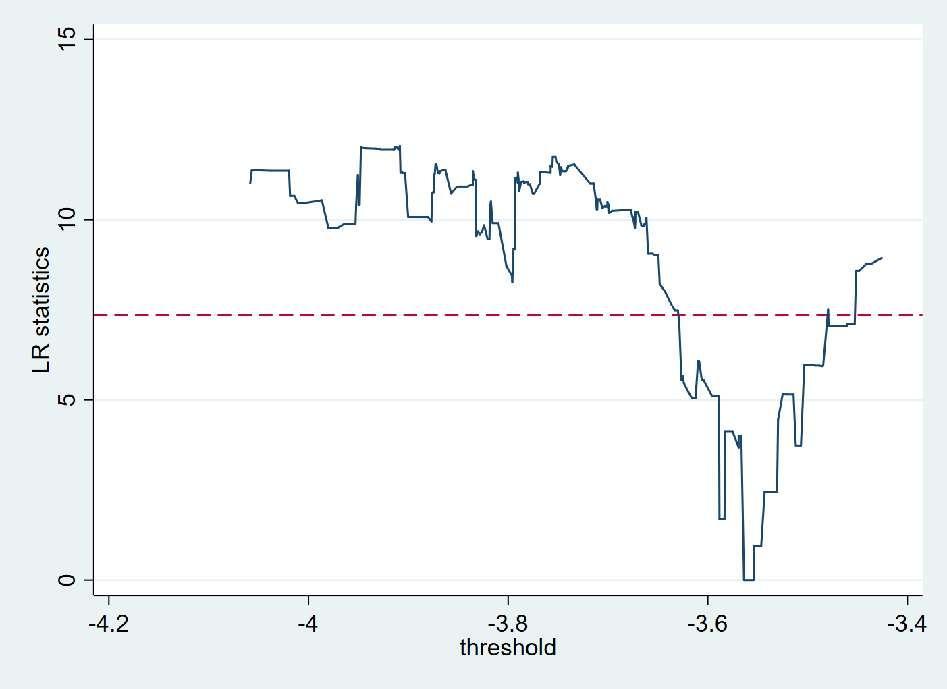


**Fig 2. Likelihood ratio function graph of financing constraints thresholds**


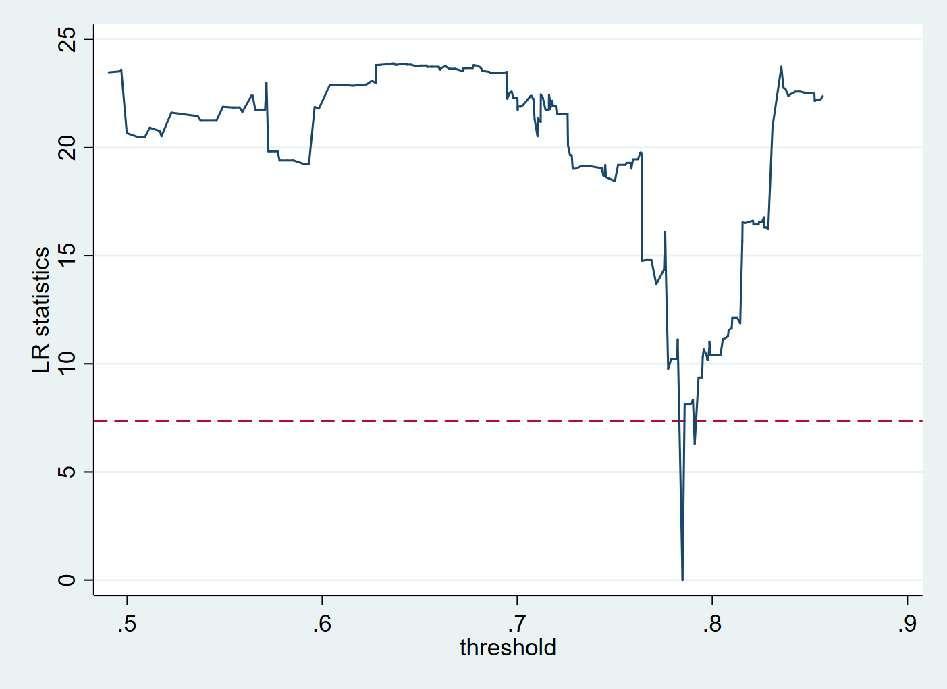


**Fig 3. Likelihood ratio function graph of operating cost thresholds**

**
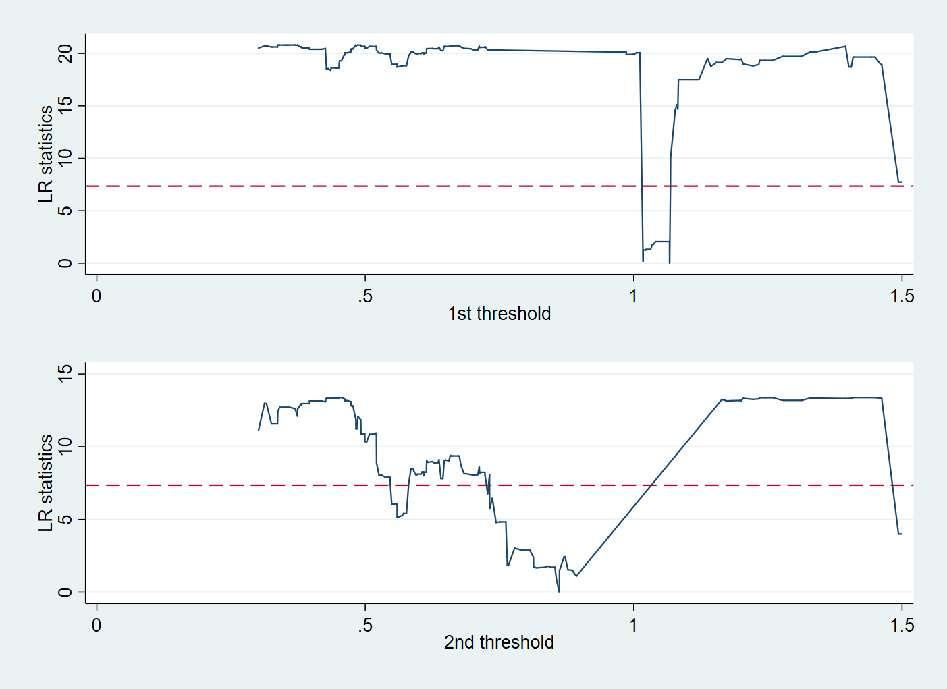
**

**Fig 4. Likelihood ratio function of the operational efficiency threshold**
